# Vertical Transmission of Extended-Spectrum, Beta-Lactamase-Producing *Enterobacteriaceae* during Preterm Delivery: A Prospective Study

**DOI:** 10.3390/microorganisms9030506

**Published:** 2021-02-27

**Authors:** Maya Frank Wolf, Raneen Abu Shqara, Karina Naskovica, Inna Amdur Zilberfarb, Inshirah Sgayer, Daniel Glikman, Hagai Rechnitzer, Vered Fleisher Sheffer, Jacob Bornstein

**Affiliations:** 1 Department of Obstetrics and Gynecology, Galilee Medical Center, Nahariya 22100, Israel; MayaW@gmc.gov.il (M.F.W.); rabushqara@gmail.com (R.A.S.); karina.naskovica@gmail.com (K.N.); innazilberfarb@gmail.com (I.A.Z.); inshirah.sg.sh@gmail.com (I.S.); 2 Azrieli Faculty of Medicine, Bar Ilan University, Safed 1311502, Israel; Daniel.glikman@biu.ac.il (D.G.); hagair@gmc.gov.il (H.R.); VeredF@gmc.gov.il (V.F.S.); 3 Clinical Microbiology Laboratory, Galilee Medical Center, Nahariya 22100, Israel; 4 Neonatal Intensive Care Unit, Galilee Medical Center, Nahariya 22100, Israel

**Keywords:** extended-spectrum, beta-lactamase-producing *Enterobacteriaceae*, *Escherichia coli*, maternal colonization, preterm birth

## Abstract

Maternal carriage and vertical transmission of extended-spectrum, beta-lactamase-producing *Enterobacteriaceae* (ESBL-E), such as *Escherichia coli*, hamper the treatment of infections, resulting in high morbidity. *E. coli* is the most frequent cause of early-onset neonatal sepsis (EOS) in preterm infants, where ESBL-E are more frequently isolated. In this prospective, case-controlled study, maternal rectovaginal ESBL-E colonization and vertical transmission to preterm infants were assessed in 160 women with preterm premature rupture of membranes (PPROM; 57.4%) or preterm labor (42.6%); additional cultures were obtained from the placenta, amnion, and umbilical cord during preterm labor. Maternal and neonatal ESBL-E-carriage rates were 17.5% and 12.9%, respectively, and the vertical-transmission rate was 50%. Maternal ESBL-E colonization among women with PPROM was 21.3%, and in women with premature labor it was 12.6%. No correlation was observed between maternal ESBL-E-colonization and previous hospitalization or antibiotic administration during pregnancy. However, a correlation was found between placental inflammation and maternal ESBL-E colonization (*p* = 0.007). ESBL-E-colonized infants were delivered at an earlier gestational age and were more likely to have complications. Thus, the high ESBL-E carriage rate in women with threatened preterm labor, without obvious risk factors for carriage, and a high vertical transmission rate, combined with a correlation between placental inflammation and ESBL-E carriage, support maternal–neonatal ESBL-E-colonization surveillance and active measures to prevent ESBL-E-related EOS.

## 1. Introduction

Global preterm birth rates have exhibited an increasing trend over the past two decades [1]. Preterm neonates who survive are at greater risk of a range of short-term and long-term morbidities [1,2,3]. Neonatal sepsis remains a major cause of morbidity and mortality during the neonatal period, despite significant advancements in perinatal care over the last few decades [4]. Group B *Streptococcus* (GBS) and *Escherichia coli* are the most common causes of all cases of neonatal early-onset sepsis (EOS) [5,6], while *Klebsiella pneumoniae* and *E. coli* are the most frequent causative organisms in most low- and middle-income countries [7,8]. In very preterm infants, the incidence of EOS is greater with *E. coli* than with GBS [9,10].

*E. coli* strains causing EOS exhibit a higher resistance to ampicillin, gentamicin, and third-generation cephalosporins in preterm infants than in term infants (93.3% vs. 48.4%, *p* < 0.01) [8,11]. Neonatal infections with extended-spectrum beta-lactamase (ESBL)-producing *Enterobacteriaceae* (ESBL-E), such as cefotaxime-resistant *E. coli*, are associated with higher mortality rates, longer neonatal intensive care unit (NICU) stays, and reduced clinical and microbiological responses [12]. Neonates could be exposed to ESBL-E during parturition (via passage through the birth canal) or before delivery (after rupturing of the amniotic membrane) [5,13]. Thus, the maternal rectovaginal ESBL-E-carriage rate has recently gained increasing attention. The reported prevalence of vaginal ESBL-E colonization during pregnancy ranges from 4.0%– 19.9% [14,15], and varies with different geographical locations, e.g., it is as high as 15%–17% in Africa versus 7% in Argentina [14,16]. The vertical-transmission rate also varies; previous data revealed a high maternal–neonatal-transmission rate of 35.7% in Norway [17], whereas other studies did not report a significant correlation between the maternal and neonatal ESBL-E status [18].

Although most cases of histological chorioamnionitis are sub-clinical in nature [19], infection may be implicated in a substantial proportion of women who undergo preterm labor [20,21,22]. The role of maternal rectovaginal ESBL-E carriage remains unknown [23], and it is unclear whether it is correlated with preterm birth. 

Very-low-birthweight (VLBW) infants are at the greatest risk of developing invasive disease, because of their compromised immunity. These infants are more likely to develop sepsis from Gram-negative organisms, including *E. coli*, rather than GBS; therefore, the greatest risk of maternal ESBL-E colonization and possible vertical transmission is for VLBW infants [5,8,9].

This study aimed to evaluate the rate of ESBL-E colonization among women in preterm labor and women with preterm premature rupture of membranes (PPROM), incidence of maternal vertical transmission, intrauterine inflammation, and the clinical significance of ESBL-E in preterm infants. We assumed that ESBL-E-colonized infants would be delivered at an earlier gestational age (GA) and develop more prematurity-related complications. The principal conclusions of this study are that women with threatened premature delivery had relatively high rates of ESBL-E rectovaginal colonization (17.5%) and vertical transmission (50%).

## 2. Materials and Methods

### 2.1. Study Description

This prospective, case-controlled study was carried out in the delivery rooms and maternal–fetal unit of a single, tertiary, university-affiliated hospital from March 2017 to December 2019. The Institutional Review Board of Galilee Medical Center approved the study (approval code 0188-16 NHR). The ClinicalTrials.gov identifier is NCT03251885. The study included women presenting with threatened preterm delivery at <37 weeks. Preterm labor was diagnosed as uterine contractions and cervical dilatation over time or PPROM. PPROM diagnosis was made based on a history of fluid leakage accompanied by pooling of amniotic fluid observed upon sterile speculum examination. If there was no clear pooling, the AmniSure ROM test (AmniSure International LLC, Boston, MA, USA), an immune-chromatography method, was used to confirm the diagnosis. Exclusion criteria were active preterm labor with cervical dilatation >6 cm, cases where an emergency cesarean section was performed owing to fetal distress, bleeding from placenta previa, acute placental abruption, and cord prolapse. Written informed consent was obtained from all participants. This study was performed in accordance with the code of ethics of the World Medical Association (Declaration of Helsinki) for experiments involving humans.

All women included in this study were hospitalized for conservative treatment, including the use of tocolysis, magnesium sulfate administration for neuroprotection before 32 weeks of gestation (if indicated), antenatal corticosteroids, and prophylactic antibiotic courses in cases of PPROM.

### 2.2. Maternal Screening

A routine maternal rectovaginal swab for GBS was obtained in cases of threatened premature labor or PPROM. In addition, another rectovaginal swab for ESBL-E was performed upon admission, according to the study protocol. A positive ESBL-E colonization result, as a single finding, was not considered an indication for antibiotic treatment. Furthermore, during preterm labor, surface swab cultures were routinely obtained from the placenta, amnion, and umbilical cord to detect colonization and infection. 

### 2.3. Neonatal Screening and Birth Cultures

Infants who were admitted to the NICU were screened for ESBL-E and included in this study. Rectal samples were obtained upon admission to the NICU to detect early colonization and implement appropriate neonatal isolation measures [17,24]. Maternal, neonatal, and placental cultures were immediately placed in Amies transport nutrient medium (COPAN, Brescia BS, Italy) and transferred to the microbiology laboratory. The results were reported within 48–72 h.

### 2.4. Identification of ESBL Bacteria in Culture and Susceptibility Tests

All specimens were cultured on CHROMagar ESBL (HY-Labs, Rehovot, Israel) growth medium and incubated for 18–24 h at 35 °C. This growth medium enables selective growth of mainly ESBL bacteria, and each bacterial species is characterized by a different colony color [24]. Final bacterial identification was made by VITEK MS systems (bioMérieux SA, Marcy-l’Étoile, France). Antibiotic susceptibility tests and final identification of the mechanism of ESBL resistance was performed by VITEK 2 systems (bioMérieux SA). The VITEK 2 expert system allows ESBL phenotype identification of *E. coli*, *Klebsiella* spp., and *Proteus* spp. This research focused on *E. coli* and *Klebsiella* spp. as they are the two most common ESBL-E. Susceptibility patterns to non-beta-lactam antimicrobials were compared in cases where ESBL-E were found in mothers and neonates.

### 2.5. Risk Factors for Maternal ESBL Carriage

We retrieved detailed patient information in terms of maternal demographics, obstetric histories, and antepartum records. Data on the hospitalization course were also collected, namely the GBS rectovaginal carriage status, urinary culture test results, administration of antibiotics during the current pregnancy, and previous hospitalizations during the current pregnancy.

### 2.6. Risk Factors for Vertical Transmission of ESBL

Data regarding antibiotic treatment during the week before delivery, PPROM, the latency period duration (defined as the period from PPROM until delivery), the mode of delivery, intrapartum fever, intrapartum antibiotic administration as indicated, birth weight (g), and the GA at delivery were obtained. After each preterm delivery, the placenta was subjected to histopathological examination for signs of inflammation showing neutrophilic infiltrate of the membrane, chorion, or subchorionic plate fibrin, scored by a specialized pathologist, as follows: grade 1, subchorionitis; grade 2, chorionitis; or grade 3, chorioamnionitis. 

### 2.7. Neonatal Outcome

The appearance, pulse, grimace, activity, and respiration (APGAR) score; cord pH; antibiotic administration; NICU admission; and complications were recorded. The major complications included the following: (a) respiratory distress syndrome (RDS), defined as progressive respiratory failure manifested by an increase in dyspnea and oxygen requirement in conjunction with a characteristic radiologic pattern (including atelectasis with aerated airways and pulmonary edema); (b) necrotizing enterocolitis (NEC), defined by abdominal distention, bilious vomiting or gastric aspirate, and rectal bleeding, along with intestinal intramural gas on abdominal X-ray; (c) intraventricular hemorrhage (IVH), diagnosed by clinical features of irritability, apnea, or seizures, and confirmed by cranial ultrasound or magnetic resonance imaging (MRI); and (d) bronchopulmonary dysplasia (BPD), a condition of chronic lung disease due to the disruption of pulmonary development, typically diagnosed when the neonate shows respiratory distress after 28 days of age or past 36 weeks post-conceptional age. The rate of ventilation support was also recorded, defined by the need for either noninvasive ventilation, such as Neopuff T-piece resuscitator and continuous positive airway pressure (CPAP), or invasive mechanical ventilation. Finally, neonatal sepsis and death rate were recorded. Neonatal outcomes were compared between the NICU infants with or without ESBL-E colonization.

### 2.8. Statistical Analysis

Continuous variables are presented as the mean ± standard deviation (SD), or as median and range values. Qualitative variables are presented as frequencies and percentages. Continuous variables were compared between the two groups using either the independent sample *t*-test or the Mann–Whitney test, based on the sample sizes of the groups and the distribution shapes of the variables. Categorical variables were analyzed using Pearson’s chi-squared test or Fisher’s exact test. To examine whether neonatal ESBL-E status was correlated with complications, a multivariate logistic regression for neonatal outcomes was performed and adjusted to GA, neonatal gender, and mode of delivery, and was based on neonatal colonization by a backward elimination regression. A multivariate linear regression model was used for NICU hospitalization length, adjusted to the above-mentioned variables. A two-tailed *p* value of <0.05 was considered significantly different. To detect neonatal morbidity, pooled ratios for RDS, NEC, IVH, and BPD were determined. Furthermore, pooled ratios for positive cultures from amniotic fluid, placentas, and umbilical cords were determined. Statistical analysis was performed by the Statistics Unit at Galilee Medical Center using IBM SPSS Statistics for Windows, version 25.0 (IBM Corp., Armonk, NY, USA).

## 3. Results

### 3.1. Characteristics of the Study Population

**Figure 1:**
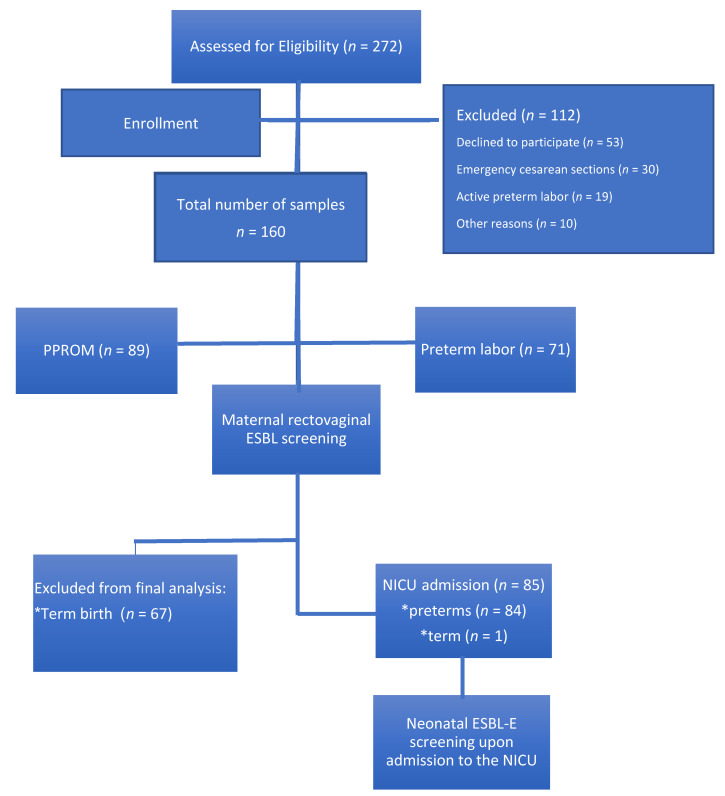
Study population. ESBL-E: extended-spectrum beta-lactamase-producing *Enterobacteriaceae*; NICU: neonatal intensive care unit; PPROM: preterm premature rupture of membranes.

In total, 160 pregnant women with threatened premature labor were recruited for the study (Figure 1); 42.6% of these patients had premature labor with uterine contractions and cervical dynamic change, whereas 57.4% had PPROM. No significant differences in the background data were observed between the two groups, except for previous preterm birth (Table 1). GA at recruitment was similar between the two groups. Of the 160 patients, 139 were screened for rectovaginal GBS at the time of admission; carriage rate was similar between both groups (Table 2).

### 3.2. Prevalence of Maternal ESBL-E Colonization and Risk Factors

**Figure 2:**
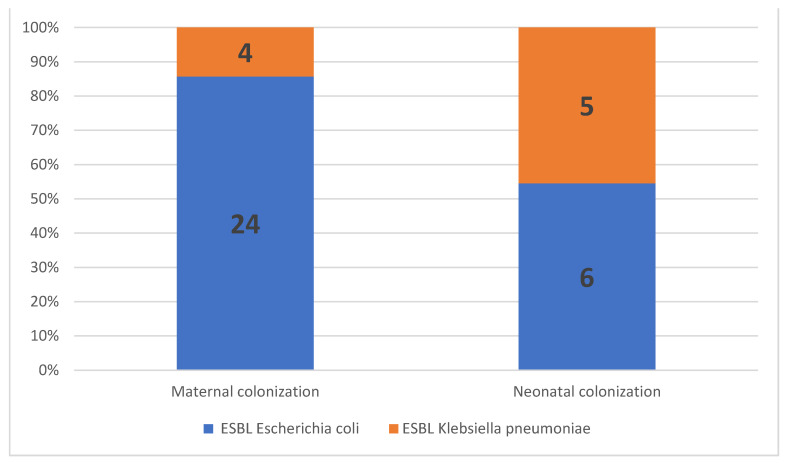
Proportion of extended-spectrum, beta-lactamase-producing *Enterobacteriaceae* (ESBL-E) isolates in maternal and neonatal colonization.

The prevalence of maternal ESBL colonization was 17.5% (*n* = 28) among all patients, 21.3% (*n* = 19) in women with PPROM, and 12.6% (*n* = 9) in women with premature labor (*p* = 0.292; Table 2). ESBL *E. coli* was isolated from most women with ESBL colonization (85.7%, *n* = 24/28), followed by ESBL *K. pneumoniae* (14.3%, *n* = 4/28; Figure 2). No significant correlations were observed between the maternal ESBL-E-colonization status and maternal baseline characteristics, including age, parity, gravidity, or previous hospitalization. In addition, no correlation was observed between the maternal ESBL-E-colonization status and the GBS-carriage status, administration of antibiotics, or urinary tract infections during pregnancy (Table 3).

### 3.3. Maternal ESBL-E Colonization and Obstetric Outcomes

In total, 160 women were included in the outcome analysis. The median GA at delivery was 35.8 (25.4–42.3) weeks, and the cesarean section rate was 29.6%. No correlation was observed between maternal ESBL-E colonization status and GA at delivery (Table 4). Eighty ESBL-E preterm birth cultures (placenta, umbilical cord, and amnion) were analyzed, and no significant differences were observed in the positive ESBL-E birth cultures between ESBL-E-colonized and non-colonized mothers (*p* = 0.062). However, in 60 placenta samples that were submitted for histopathological examination, a correlation was observed between placental inflammation signs and maternal ESBL-E colonization status (*p* = 0.007; Table 4).

### 3.4. Neonatal Outcomes

Eighty-five neonates (53.1%) were admitted to the NICU; among them, 84 were preterms (GA < 37) and one was delivered at term (GA = 39, admitted due to fever at birth, negative for ESBL-E). Of the 85, 18.9% were born to ESBL-E-colonized mothers. The overall ESBL-E-colonization rate among preterm infants was 13% (11/84). ESBL *E. coli* was isolated from 6 out of 11 preterm infants (55%), and ESBL *K. pneumoniae* was isolated from the remaining five preterm infants (45%). Of note, ESBL *K. pneumoniae* colonization was more prevalent in the preterm infants than in the mothers (Figure 2). 

ESBL-E-colonized neonates were born at an earlier GA (median = 30.3 (25.4–34.0) vs. 34 (26.6–36.9) weeks, *p* = 0.006) and had a relatively lower birthweight (1502.4 ± 498.9 g vs. 2032.5 ± 692.6 g, *p* = 0.009) than neonates without ESBL-E colonization. No correlations were observed between the mode of delivery, antepartum or intrapartum antibiotic administration, latency period, presence of PPROM, intrapartum fever, and risk of neonatal ESBL-E colonization at the time of NICU admission (Table 5). Also, no correlation was observed between ESBL-E status in NICU-admitted neonates and the 5 min APGAR score (<7) or cord pH (<7.10) (Table 4 and Table 5).

Univariate analysis revealed a correlation between hospitalization duration, antibiotic administration in the NICU, and neonatal complications with neonatal ESBL-E colonization status (*p* = 0.049, *p* = 0.03, and *p* = 0.023, respectively); no difference was observed in the need for ventilation support or perinatal mortality rate with respect to neonatal ESBL-E status (Table 5). Multivariate analysis, used to evaluate the correlation between neonatal ESBL-E status and neonatal outcomes, adjusted for delivery week, gender, and mode of delivery, revealed that delivery week was uniformly correlated with all outcomes. Cesarean sections increased NICU hospitalization length (Table 6). Of note, one case of neonatal death occurred following ESBL-E vertical transmission in a preterm born at 28.2 weeks of gestation, owing to PPROM. The neonate received empiric antibiotic treatment with gentamicin and ampicillin, but presented with ESBL-E sepsis and subsequent peritonitis. Although the antibiotic was switched to meropenem, the infant did not survive.

### 3.5. Vertical Transmission of ESBL

**Figure 3:**
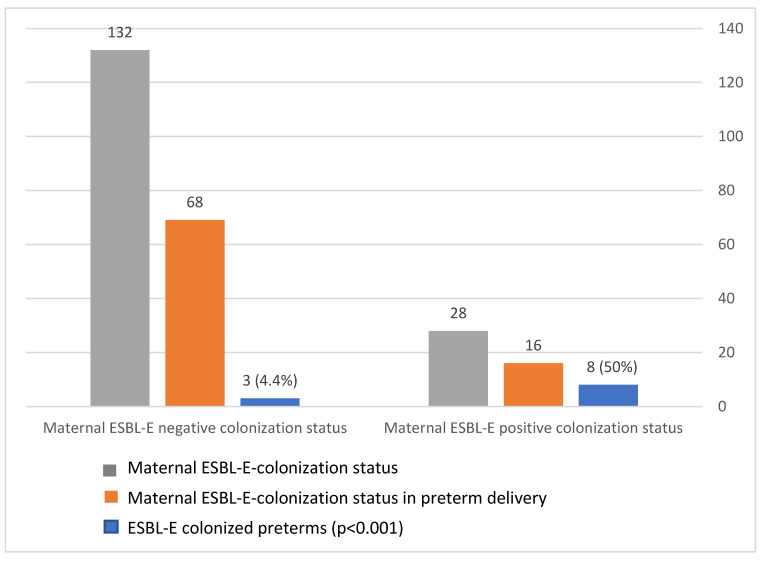
Extended-spectrum, beta-lactamase-producing *Enterobacteriaceae* (ESBL-E) transmission rate during preterm delivery.

The maternal–neonatal transmission rate was 50% (8/16, *p* < 0.001; Figure 3). Furthermore, 72.2% of ESBL-colonized preterm infants were born to an ESBL-colonized mother, whereas only 10% of ESBL-negative neonates were born to ESBL-colonized mothers (*p* < 0.001). Thus, maternal ESBL colonization is correlated with neonatal positive colonization status. We also observed identical non-beta-lactam antimicrobial susceptibility (to gentamicin, amikacin, trimethoprim/sulfa, and ciprofloxacin) in cases of paired maternal–neonatal ESBL-E colonization.

## 4. Discussion

### 4.1. Main Findings

The primary findings of this study are the ESBL-E rectovaginal colonization rates in women with threatened premature delivery (17.5%) and the high vertical-transmission rates (50%). The ESBL-E-carriage rate of our study population was comparable to that reported in Africa [14,16] and substantially higher than that observed in high- and middle-income countries, e.g., Norway (2.9%) [17] and Argentina (5.4%) [15], respectively. We also observed a slightly higher maternal–neonatal transmission rate than previously reported (50% vs. 35%) [17,25]. These findings suggest that maternal ESBL-E colonization constitutes a substantial risk for infant colonization.

Although a positive correlation was previously observed between maternal ESBL-E colonization and urinary tract infections during pregnancy, antibiotic administration, and hospitalization [26], the present results do not support these findings, and no correlation between maternal ESBL-E-colonization status and urinary tract infection during pregnancy was observed. Nevertheless, our findings of a high ESBL-E carriage rate in women with threatened preterm labor, without obvious risk factors for carriage, and a high vertical transmission rate support maternal–neonatal ESBL-E-colonization surveillance in threatened preterm labor.

### 4.2. Ascending Infection and Placental Involvement

We observed a significant correlation between maternal ESBL-E colonization status and signs of histopathologic placental inflammation; however, no significant correlation was observed between maternal ESBL-E colonization and the pooled ratio of ESBL-E isolates from placenta, umbilical cord, and amnion cultures. These findings suggest that maternal ESBL-E colonization predisposes individuals to ascending infection and placental involvement. Ascending infection has been implicated as a cause for preterm delivery, as it promotes multiple physiological events, including increased levels of pro-inflammatory cytokines, membrane rupture, cervical ripening, and uterine contraction [27]. In this study, ESBL-E-colonized preterm infants were more likely to be born at an earlier GA. Of note, neonatal screening was conducted on the first day of NICU admission; hence, it appears that the preterm neonates acquired ESBL-E through the birth canal or more likely pre-delivery [28], resulting in a sub-clinical infection that may have initiated the preterm birth. Previous data support the vertical-transmission theory by showing that mother–neonate pairs are colonized with identical ESBL-E, based on species identification and antibiograms with 70% shared molecular-fingerprinting patterns [25,29,30].

### 4.3. Clinical Outcomes

The correlation between neonatal ESBL-E colonization and RDS or the need for ventilation support has been previously reported [31]. Multivariate analysis revealed that the primary factor affecting newborn complications was GA at birth; we did not observe a significant association between neonatal ESBL-E status and neonatal complications and hospitalization length in the NICU, probably because of the small sample size. With adjustment for confounders, we did not observe a correlation between neonatal ESBL-E status and the need for ventilation or complications. Previous studies have indicated a high fatality rate in neonatal ESBL-E infections, which was most likely due to inadequate antibiotic treatment [28]. Although we did not observe a higher fatality rate in ESBL-E-colonized preterm infants, the one case of neonatal mortality occurred in an ESBL-E-colonized newborn with ESBL-E sepsis.

### 4.4. Strengths and Limitations

There are some limitations associated with the study. Specifically, we employed a relatively small sample size, and only NICU-admitted infants were screened for ESBL-E. Therefore, term infants that were not admitted to the NICU but had potential ESBL-E colonization were excluded from the final analysis. Another limitation is the inability to prove direct vertical transmission, since molecular typing of the ESBL-E-positive isolates was not performed, although we observed identical antimicrobial susceptibility patterns in cases of maternal–neonatal ESBL-E colonization. In addition, *Mycoplasma* and *Ureaplasma* colonization rates are not reported in this study, since these species are not routinely cultured at the bacteriology laboratory of our institute. The coexistence of ESBL-E and *Mycoplasma* could be a possible confounder in the interpretation of the correlation between ESBL-E and placental inflammation. Despite these limitations, our study has several unique strengths, in that it was a prospective study with controlled groups of ESBL-E-colonized and non-colonized mothers and preterm infants. This study allowed us to compare risk factors and outcomes. To the best of our knowledge, this is the first study to analyze potential correlations between maternal ESBL-E-colonization status and cultures obtained during delivery from the placenta, amnion, and umbilical cord.

### 4.5. Implications

Antenatal screening for GBS carriage is a routine part of antenatal care in most developed countries, including the United States [32]. The prevalence of rectovaginal GBS colonization in pregnant women ranges between 10% and 30% [33,34]. Although *E. coli* (including ESBL *E. coli*) is currently the most common pathogen in preterm infants, and is associated with a higher mortality rate than that of GBS [14,16,25,34], no universal screening test has been recommended for detecting ESBL-E carriage. In addition, although intrapartum GBS-targeting prophylactic antibiotics are usually administered in preterm deliveries in countries where routine screening for GBS is not performed, these antibiotics do not exhibit activity against ESBL-E [32,35]. This factor may emphasize the need for universal surveillance for ESBL-E among women with threatened premature labor.

Moreover, the relatively high prevalence of ESBL-E colonization among women with threatened premature delivery, no obvious risk factors for colonization, and high ESBL-E-transmission rate observed in this study is worrisome. The greatest risk of maternal ESBL-E colonization and possible vertical transmission is for VLBW infants, since these infants are more likely to develop sepsis from Gram-negative organisms [5,8,9].

The correlation between placental inflammation and ESBL-E carriage and the identical non-beta-lactam antimicrobial susceptibility observed in paired maternal–neonatal ESBL-E colonization supports maternal–neonatal ESBL-E-colonization surveillance and active measures to prevent ESBL-E-related EOS. Additional studies are required to further explore the effect of maternal ESBL-E colonization on short- and long-term neonatal health outcomes, and to evaluate the efficacies of various intrapartum antibiotic regimens in ESBL-E-colonized mothers.

## Figures and Tables

**Table 1:** Baseline characteristics of women participating in the study.

Baseline Characteristic		Cohort (*n* = 160)	Preterm Labor (*n* = 71)	PPROM (*n* = 89)	*p*-Value
Maternal Age (y)	Mean ± SD	29.6 ± 6.3	28.8 ± 6.2	29.9 ± 6.4	0.29
Parity	Median (range)	2 (1–10)	2 (1–5)	2 (1–10)	0.295
Gravidity	Median (range)	2 (1–14)	2 (1–7)	2 (1–14)	0.191
Previous Preterm Birth		36 (22.5%)	21 (29.6%)	15 (17%)	0.033
Cervical Cerclage in Situ		11 (6.8%)	3 (4.2%)	8 (8.9%)	0.358
Maternal Diabetes		16 (10.0%)	9 (12.7%)	7 (7.9%)	0.284
Multiple Gestations		7 (4.5%)	5 (7.0%)	2 (2.2%)	0.132

PPROM: preterm premature rupture of membranes; SD: standard deviation.

**Table 2:** Obstetric outcomes stratified by PPROM and preterm labor.

Variable		Cohort (*n* = 160)	Preterm Labor (*n* = 71)	PPROM (*n* = 89)	*p*-Value
ESBL-E Status		28 (%5.17)	9 (12.6%)	19 (21.3%)	0.292
Sampling Week	Median (range)	33.64 (24.1–36.9)	33.57 (24.9–36.9)	33.71 (24.1–36.9)	0.481
GBS Carriage Status	*n* =139	22/139 (15.8%)	11/59 (18.6%)	11/80 (13.8%)	0.485
Delivery Week	Median (range)	35.8 (25.4–42.3)	37.0 (29.1–40.6)	34.6 (25.4–42.3)	<0.001
Delivery Mode among Final Sample	Cesarean	45 (28.1%)	13 (18.3%)	32 (36.0%)	0.032

ESBL-E: extended-spectrum, beta-lactamase-producing *Enterobacteriaceae*; GBS: group B *Streptococcus*; PPROM: preterm premature rupture of membranes.

**Table 3:** Association between maternal characteristics and ESBL-E colonization status.

Variable		Cohort (*n* = 160)	Negative Maternal ESBL-E-Colonization (*n* = 132)	Positive Maternal ESBL-E Colonization (*n* = 28)	*p*-Value
Sampling Week	Mean ± SD	32.63 ± 3.45	32.75 ± 3.42	32.09 ± 3.61	0.356
Maternal Age (years)	Mean ± SD	29.61 ± 6.36	29.43 ± 6.02	30.54 ± 7.93	0.251
Gravidity	Mean ± SD	2.55 ±1.86	2.48 ± 1.65	2.88 ± 2.70	0.301
Parity	Mean ± SD	1.95 ± 1.29	1.88 ± 1.08	2.31 ± 2.015	0.253
Hospital Admissions	Median(range)	0.54 ± 0.78	0.56 ± 0.79	0.46 ± 0.71	0.331
Positive GBS Status	*n* = 139	22/139 (15.8%)	19/117 (16.2%)	3/22 (13.6%)	1.00
UTI in Current Pregnancy		19 (11.8%)	14 (10.6%)	5 (17.9%)	0.332
Antibiotic Administration (Prior to Sampling)		12 (7.5%)	8 (6)	4 (14.2%)	0.110

ESBL-E: extended-spectrum, beta-lactamase-producing *Enterobacteriaceae*; GBS: group B *Streptococcus*; UTI: urinary tract infection.

**Table 4:** Association between maternal ESBL-E colonization and perinatal outcomes.

Variable		Cohort (*n* = 160)	Negative Maternal ESBL-E-Colonization (*n* = 132)	Positive Maternal ESBL-E Colonization (*n* = 28)	*p*-Value
Delivery Week	Median (range)	35.8 (25.4–42.3)	35.9 (26.6–42.3)	35.4 (25.4–40.0)	0.659
Delivery Mode	Cesarean	45 (28.1%)	36 (27.3%)	9 (32.1%)	0.60
Placental Inflammation Signs	*n* = 61	17/61 (27.9%)	10/50 (20%)	7/11 (63.6%)	0.007
ESBL-E Isolates (Placenta, Cord, Amnion)	Pooled ratio (*n* = 80)	5/80 (6.3%)	2/63 (3.2%)	3/17 (17.6%)	0.062
**Perinatal Outcome**					
NICU Admission	*n* = 85	85 (53.1%)	69/132 (52.2%)	16/28 (57.1%)	0.835
Positive Neonatal ESBL-E-Colonization (at Admission)	*n* = 85	11/85 (12.9%)	3/69 (4.3%)	8/16 (50%)	<0.001

ESBL-E: extended-spectrum, beta-lactamase-producing *Enterobacteriaceae*; NICU: neonatal intensive care unit; SD: standard deviation.

**Table 5:** Risk factors and outcomes of ESBL-E colonization among neonates in the NICU.

Risk Factor		NICU Admission (*n* = 85)	Negative Neonatal ESBL-E-Colonization (*n* = 74)	Positive Neonatal ESBL-E-Colonization (*n* = 11)	*p*-Value
Maternal ESBL Colonization		16 (18.8%)	8 (10.8%)	8 (72.7%)	<0.001
Neonatal Gender	Male Female	56% 44%	52.1% 47.9%	81.8% 18.2%	0.102
Delivery Mode	Cesarean	38 (44.7%)	34 (45.9%)	4 (36.4%)	0.747
	Vaginal	47 (55.3%)	40 (54.1%)	7 (63.6%)	
Delivery Week (Weeks) *	Median (range)	33.6 (25.4–36.9)	34 (26.6–36.9)	30.3 (25.4–34.0)	0.007
Antepartum Antibiotics (7-Day Interval)		33 (38.8%)	31 (41.9%)	2 (18.2%)	0.190
Intrapartum Antibiotics		79 (94.0%)	70 (94.6%)	9 (90.0%)	0.478
Intrapartum Fever		5 (5.9%)	5 (6.8%)	0 (0%)	0.491
Latency Period (days)	Median (range)	2 (0–60)	2 (0–52)	3.5 (0–60)	0.974
PROM		64 (75.3%)	54 (73%)	10 (90.9%)	0.279
**Outcomes**					
Birthweight (g) *	Mean ± SD	1966.7 ± 628.1	2032.5 ± 692.6	1502.4 ± 498.9	0.011
NICU Hospitalization Length (days)	Median (range)	18.0 (21–04)	15.5 (2–104)	23.0 (6–98)	0.049
Antibiotic Administration in NICU (%)		61 (71.8%)	51 (68.9%)	10 (90.0%)	0.168
Antibiotic Administration in NICU (days)	Mean ± SD	2.52 ± 2.48	0.59 ± 2.12	6.00 ± 4.30	0.03
Ventilation Support		39 (45.9%)	31 (41.9%)	8 (72.2%)	0.056
Neonatal Morbidity Pooled Ratio in NICU		34 (40%)	26 (35.1%)	8 (72.7%)	0.023

ESBL-E: extended-spectrum, beta-lactamase-producing *Enterobacteriaceae*; NICU: neonatal intensive care unit; PPROM: preterm premature rupture of membranes; SD: standard deviation. * Term neonate (1/85) was excluded from the analysis of delivery week and birthweight.

**Table 6:** Multivariate regression: correlation between variables and neonatal outcome in NICU.

Neonatal Complications (*R*^2^ = 46.3%)	*p*-Value	Odds Ratio	95% CI Lower Limit	95% CI Upper Limit
Positive Neonatal ESBL-E-Colonization	0.124	3.656	0.7000	18.984
Delivery Week	*p* < 0.001	0.0170	0.002	0.164
Delivery Mode (Cesarean vs. Vaginal)	0.647	1.302	0.420	4.034
Gender (Female vs. Male)	0.076	0.322	0.092	1.125
**Need for Ventilation** **(*R*^2^ = 58.0%)**	***p*-Value**	**Odds Ratio**	**95% CI** **Lower limit**	**95% CI** **Upper**
Positive Neonatal ESBL-E-Colonization (Positive versus Negative)			0.1990	9.082
Delivery Mode (Cesarean versus Vaginal)	0.776	1.208	0.329	4.432
Delivery Week	*p* < 0.001	0.450	0.313	0.646
Gender (Female versus Male)	0.363	0.559	0.160	1.955
**NICU Hospitalization Length** **(*R*^2^ = 60.9%)**	***p*-Value**	**B-Standardized coefficients**		
Positive Neonatal ESBL-E-Colonization	0.179	0.097		
Delivery Mode (Cesarean versus Vaginal)	*p* < 0.001	0.291		
Delivery Week	*p* < 0.001	−0.648		
Gender (Female versus Male)	0.190	−0.093		

ESBL-E: extended-spectrum, beta-lactamase-producing *Enterobacteriaceae*; NICU: neonatal intensive care unit.

## Data Availability

Not applicable.

## Abbreviations

ESBL-E	extended-spectrum beta-lactamase producing Enterobacteriaceae.
EOS early	onset neonatal sepsis
PPROM	preterm premature rupture of membranes
ESBL	extended-spectrum beta-lactamase
GBS	Group B Streptococcus
*E. coli*	*Escherichia coli*
NICU	neonatal intensive care unit
VLBW	very-low-birthweight
GA	gestational age
APGAR score	appearance, pulse, grimace, activity, and respiration
RDS	respiratory distress syndrome
NEC	necrotizing enterocolitis
IVH	intraventricular hemorrhage
MRI	magnetic resonance imaging
BPD	bronchopulmonary dysplasia
CPAP	continuous positive airway pressure
SD	standard deviation
